# Robotic Pancreaticoduodenectomy Using the Right Posterior Superior Mesenteric Artery Approach

**DOI:** 10.1007/s11605-023-05806-6

**Published:** 2023-08-14

**Authors:** Kosei Takagi, Yuzo Umeda, Tomokazu Fuji, Kazuya Yasui, Toshiyoshi Fujiwara

**Affiliations:** Department of Gastroenterological Surgery, Okayama University Graduate School of Medicine, Dentistry, and Pharmaceutical Sciences, 2-5-1 Shikata-cho, Kita-ku, Okayama, 700-8558 Japan

**Keywords:** Pancreatoduodenectomy, Robotic surgery, Surgical approach, Superior mesenteric artery

## Introduction

Various surgical strategies to the superior mesenteric artery (SMA) have been reported in robotic pancreaticoduodenectomy (RPD).^1^ Of several approaches to the SMA during RPD,^2^ the right posterior SMA approach could be optional and helpful in selected patients; however, only a few studies have described the right posterior approach in RPD. Herein, we demonstrate the technical aspects of the right posterior SMA approach for RPD (supporting video 1).

### Surgical Techniques of the Right Posterior SMA Approach in RPD

Our standard protocol for RPD includes two-surgeon technique which does rely on a skilled patient-side assistant operating the vascular clips and ultrasonic shears (Ligasure).^2,3^ Surgical resection starts with the Kocher maneuver until the anterior wall of the inferior vena cava and the left renal vein are exposed. Once the Treitz ligament is divided extensively, the jejunum is pulled into the right upper quadrant space and transected with a linear stapler. After opening the gastrohepatic ligament, the stomach is divided with a linear stapler for the subtotal stomach-preserving technique. Regarding the dissection of the hepatoduodenal ligament, the gastroduodenal artery is identified and divided with a vascular stapler. The common bile duct is identified and transected. The pancreas is dissected on the superior mesenteric vein (SMV), but is not divided at this step.

**Fig. 1:**
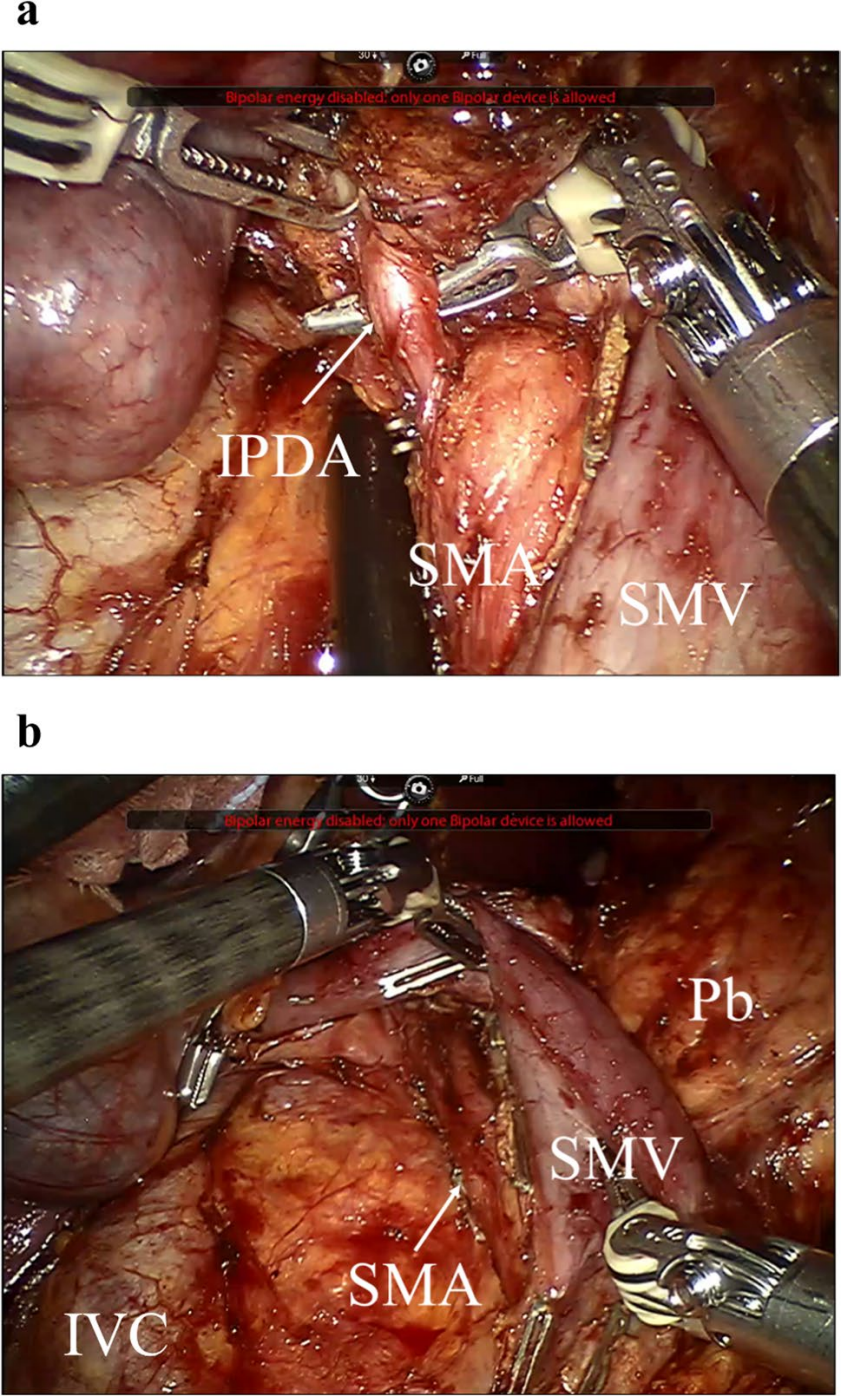
The right posterior SMA approach during robotic pancreaticoduodenectomy. During the uncinate process dissection, the posterior, right, and anterior sides of the SMA are dissected, and small branches from the mesenteric vessels, including the IPDA, are divided (**a**). Using the right posterior SMA approach, the pancreatic body is mobilized leftwards in a unique view (**b**). Finally, the pancreatic neck is transected on the SMV, and the specimen is resected. SMA, superior mesenteric artery; SMV, superior mesenteric vein; IPDA, inferior pancreaticoduodenal artery; IVC, inferior vena cava; Pb, pancreatic body

The pancreatic head is mobilized, and the right posterior SMA approach is applied. Regarding the uncinate process dissection, the posterior, right, and anterior sides of the SMA are dissected in a cranial direction to detach the pancreatic neck from the mesenteric vessels. Small branches from the mesenteric vessels, including the inferior pancreaticoduodenal artery, should be clipped and divided (Fig. 1a). The pancreatic head is pulled leftwards, and the anterior side wall of the SMA is revealed. Following the dissection of the perivascular connective tissues along the vascular axis, the uncinate process is completely detached. Using the right posterior SMA approach, the pancreatic body is mobilized leftwards in a unique view (Fig. 1b). Finally, the pancreatic neck is transected on the SMV, and the specimen is resected.

## Discussion

The present study describes the right posterior SMA approach during RPD. The right posterior approach allows dissection of the pancreatic head along the mesenteric vessels with the unique view from the caudal side, as well as easy mobilization of the pancreatic head toward the pancreatic body prior to the division of the pancreas. Moreover, division of the pancreas at the last step can prevent intraabdominal contamination by the pancreatic juice.

A previous review has reported that an indication of the right posterior approach would be for posteromedial tumors in pancreatic head, and periampullary tumors extending from body and head.^4^ The advantages of the right posterior approach included early identification of SMA and SMV involvement, identification of replaced right hepatic artery, and adequate retropancreatic lymphadenectomy.^4^ In contrast, there are technical difficulties in patients with peripancreatic inflammation as well as adhesions around the pancreatic head.

## Conclusions

The right posterior SMA approach in RPD can be optional among various surgical strategies to the SMA. Surgeons should choose the best approach according to demographic, anatomical, and oncological factors.

### Supplementary Information

ESM 1 Video 1 Robotic pancreaticoduodenectomy using the right posterior superior mesenteric artery approach
